# Attentional Bias in Alcohol and Cannabis Use Disorder Outpatients as Indexed by an Odd-One-Out Visual Search Task: Evidence for Speeded Detection of Substance Cues but Not for Heightened Distraction

**DOI:** 10.3389/fpsyg.2021.626326

**Published:** 2021-02-15

**Authors:** Janika Heitmann, Peter J. de Jong

**Affiliations:** ^1^Verslavingszorg Noord Nederland, Groningen, Netherlands; ^2^Department of Clinical Psychology and Experimental Psychopathology, University of Groningen, Groningen, Netherlands

**Keywords:** attentional bias, visual search, substance use disorder, alcohol use disorder, cannabis use disorder, speeded detection, increased distraction

## Abstract

Current cognitive models of addiction imply that speeded detection and increased distraction from substance cues might both independently contribute to the persistence of addictive behavior. Speeded detection might lower the threshold for experiencing craving, whereas increased distraction might further increase the probability of entering a bias-craving-bias cycle, thereby lowering the threshold for repeated substance use. This study was designed to examine whether indeed both attentional processes are involved in substance use disorders. Both attentional processes were indexed by an Odd-One-Out visual search task in individuals diagnosed with alcohol use disorder (AUD; *n* = 63) and cannabis use disorder (CUD; *n* = 28). To test whether the detection and/or the distraction component are characteristic for AUD and CUD, their indices were compared with matched individuals without these diagnoses (respectively, *n* = 63 and *n* = 28). Individuals with CUD showed speeded detection of cannabis cues; the difference in detection between AUD and the comparison group remained inconclusive. Neither the AUD nor the CUD group showed more distraction than the comparison groups. The sample size of the CUD group was relatively small. In addition, participants made relatively many errors in the attentional bias (AB) task, which might have lowered its sensitivity to detect ABs. The current study provided no support for the proposed role of increased distraction in CUD and AUD. The findings did, however, provide support for the view that speeded detection might be involved in CUD. Although a similar trend was evident for AUD, the evidence was weak and remained therefore inconclusive.

## Introduction

Current cognitive models of addiction point to the relevance of heightened attentional capture of substance-relevant cues in the persistence of addictive behavior (Wiers et al., 2007; Gladwin and Figner, 2015). That is, individuals diagnosed with substance use disorders may show biased selective attention toward cues that are related to the use of substances which in turn may contribute to the development of craving (Franken, 2003; Field et al., 2009). Accordingly, people may enter a self-reinforcing bias-craving-bias cycle, lowering the threshold for repeated and regular substance use. However, recent reviews have pointed to the fact that the proposed role of attentional bias (AB) in addiction is not consistently supported by the empirical evidence (see for example Christiansen et al., 2015; Field et al., 2016).

One factor that has been discussed to complicate the empirical process of investigating the role of AB in addiction is the configuration of current tasks that have been used to measure AB (Field and Cox, 2008). First, most assessment tasks, such as the often used visual probe task (MacLeod et al., 1986), but also more recently developed reaction time tasks such as the attentional cueing task (Garland et al., 2012), the flicker change blindness paradigm (Jones et al., 2006), or the attentional blink task (Brown et al., 2018) do not allow to directly differentiate between initial orientation of attention toward a cue (thereby supporting speeded detection) and the difficulty of redirecting attention away from this cue (resulting in increased distraction; Posner, 1980; Posner and Petersen, 1990; Grafton and MacLeod, 2014). These tasks tend to deliver one overall index of AB in which detection and distraction processes are intertwined (cf. Grafton and MacLeod, 2014). Other measurement issues, which complicate differentiating between attentional detection and distraction, also yield for eye-tracking based measures of overt attention within the context of free viewing tasks in which persons’ spontaneous viewing patterns are captured (e.g., Pennington et al., 2019; Soleymani et al., 2020). Given the absence of task instructions that require to attend or look away from the substance-relevant stimuli, neither of the two processes is required to successfully complete the task. However, as suggested by studies in the field of anxiety and eating research, both cognitive mechanisms might be independently (and differentially) involved in the persistence of disorders (e.g., Grafton and MacLeod, 2014; Jonker et al., 2019a), which in turn might have relevant implications for treatment. This points to the importance of further investigating the role of AB in addiction by using measurement procedures that allow to compute separate indices of speeded detection and increased distraction (cf. Jonker et al., 2019b).

Second, it has been argued that it is important that AB assessment tasks adequately model the key features of contexts that are relevant for real-life substance use behavior (Pennington et al., 2019). Given that these contexts are likely to consist of a large variety of stimuli (e.g., imagine entering a supermarket and being confronted with many items including many different alcoholic drinks), is seems important to also use tasks with a sufficiently complex stimulus configuration. Only using a maximum of two stimuli within each trial, as it is the case with one of the most often used tasks – the visual probe task – seems to fail the ecological validity and therefore may hinder the ability to generalize study findings to real-life substance use behavior (Pennington et al., 2019). One way to increase the ecological validity of a task is by increasing the number of presented stimuli within each trial, so that the number of stimuli is more in line with the number of stimuli someone faces in a real-life substance use situation. Further, and in line with the previous point, a complex task configuration seems also essential to sufficiently challenge the attentional system (Hertel and Mathews, 2011). That is, previous work has revealed that the strength of cognitive biases may depend on the extent to which cognitive systems are challenged (e.g., Evans et al., 2011).

To arrive at more final conclusions with regard to the relevance of AB in substance use disorder it would therefore be important to use an assessment task that not only can differentiate between speeded detection and distraction, but also is characterized by a complex stimulus configuration. One promising task that meets these requirements is the so-called Odd-One-Out task (OOOT; Rinck et al., 2005), which for example has been successfully applied to study AB in the context of anxiety disorders (de Voogd et al., 2014), and unsuccessful dieting (Jonker et al., 2019b). During this visual search task, participants are presented with a series of stimulus matrices, and instructed to identify whether all stimuli belong to the same category of images or whether one stimulus is different from the others (i.e., an odd-one-out). The task includes one category of stimuli that is disorder-relevant, and two stimulus categories that are disorder-irrelevant. As a result, the OOOT includes trials in which (1) one disorder-relevant stimulus is presented among disorder-irrelevant distractors, (2) one disorder-irrelevant stimulus is presented among disorder-relevant distractors, and (3) one disorder-irrelevant stimulus is presented among another category of disorder-irrelevant distractors. The latter trial type, which does not include any disorder-relevant cue, allows for the computation of a personal baseline of how long it generally takes to find a neutral target among neutral distractors. This makes it possible to calculate separate indices for speeded detection and distraction by contrasting this neutral trial type with the two trial types which do include disorder-relevant stimuli (either as a target or as distractors).

Within the context of addiction research, thus far only two studies tested the OOOT as an assessment task to index AB toward substance-relevant cues. The first study using the OOOT focused on AB for smoking-relevant stimuli and found no support for the relevance of distinguishing between speeded detection and increased distraction in substance use, as no difference was found in either detection or distraction bias between non-smokers and heavy smokers (Oliver and Drobes, 2012). The second study, however, did provide evidence pointing to the relevance of differentiating between both biases, and showed that specifically increased distraction was related to alcohol consumption in a student sample (Heitmann et al., 2020). Given the apparent inconsistency of these findings, and the restriction of both studies to non-clinical samples, the current study aimed to further investigate the proposed relevance of speeded detection and increased distraction in addiction by focusing on a treatment-seeking sample of clinically diagnosed individuals with substance use disorder. We choose to include participants diagnosed with alcohol use disorder (AUD) and cannabis use disorder (CUD), as these two diagnoses constitute the largest group of patients in addiction care in the Netherlands (Van Laar and Van Gestel, 2017). To contrast the results of these two clinical samples, their AB indices were compared with two age and gender matched samples without a history of either AUD or CUD. In short, the aim of the current study was to examine to what extent individuals diagnosed with AUD or CUD were characterized by AB, as indicated by speeded detection of and/or increased distraction by substance-relevant stimuli, as measured with the OOOT.

## Materials and Methods

### Participants

The clinical samples that were included in the current study were recruited in the context of a multicenter randomized control trial (see Heitmann et al., 2017; Netherlands Trial Register NTR5497), that was designed to test the efficacy of an AB modification training as an add-on intervention to regular treatment for substance use disorder. Participants of the clinical samples were outpatients who were treated for AUD or CUD in Dutch addiction care. The two comparison groups consisted of participants from the community who had no history of treatment for AUD or CUD, and were at the moment of data collection not in need for treatment regarding their alcohol/cannabis use. For the current study, we originally planned to include 128 patients diagnosed with AUD or CUD (50:50) from the baseline assessment of the clinical trial, and additionally 128 matched participants without these diagnoses. However, due to various unforeseen problems (e.g., massive restructuring within participating treatment centers) fewer participants could be included in the clinical trial. Therefore, in the current study the number of included patients diagnosed with CUD is limited. Our final sample consisted of 63 patients diagnosed with AUD (i.e., alcohol group; 60.3% male, *M*_*age*_ = 49.86, SD_*age*_ = 12.34, age range: 25–69 years), and 63 adults without this diagnosis (i.e., alcohol comparison group; 55.6% male, *M*_*age*_ = 48.67, SD_*age*_ = 13.49, age range: 18–70 years). Further, this study included 28 patients diagnosed with CUD (i.e., cannabis group; 75.0% male, *M*_*age*_ = 31.21, SD_*age*_ = 7.32, age range: 20–54 years), and 28 adults without this diagnosis (i.e., cannabis comparison group; 64.3% male, *M*_*age*_ = 32.82, SD_*age*_ = 8.71, age range: 21–54 years). The alcohol group and the alcohol comparison group were comparable with regard to age and gender [*t*_*age*_ (124) = 0.52, *p* = 0.606; χ_*gender*_(1) = 0.293, *p* = 0.588], and this also yields for the cannabis group and the cannabis comparison group [*t*_*age*_ (54) = −0.75, *p* = 0.458; χ_*gender*_(1) = 0.760, *p* = 0.383].

### Material

#### Self-Report Measures

##### Demographics

Age, gender, marital status, and level of education were collected as descriptive sociodemographic information.

##### Alcohol and cannabis use

The frequency of alcohol and cannabis use, as well as the quantity of used alcohol, were assessed using the Measurements in Addiction for Triage and Evaluation Questionnaire (MATE-Q; Schippers and Broekman, 2014). To establish the frequency of use, participants were asked to indicate on how many of the past 30 days they consumed either alcohol or cannabis. The quantity of used alcohol was assessed by the number of standard units of alcohol consumed on a typical day of the week (i.e., Monday, Tuesday, Wednesday, etc.). Based on the answers the amount of used standard units of the past 30 days were calculated by multiplying the answers by four. The quantity of consumed cannabis was not assessed, since the calculation of standard units for cannabis is virtually impossible (e.g., because cannabis from different origins contains different amounts of tetrahydrocannabinol).

#### Behavioral Measure

**FIGURE 1:**
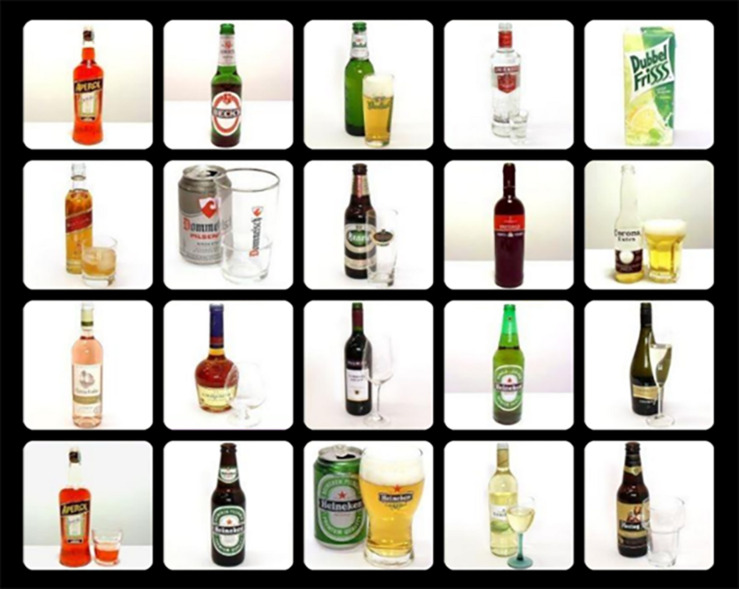
Example of an alcohol distractors trial of the OOOT.

**FIGURE 2:**
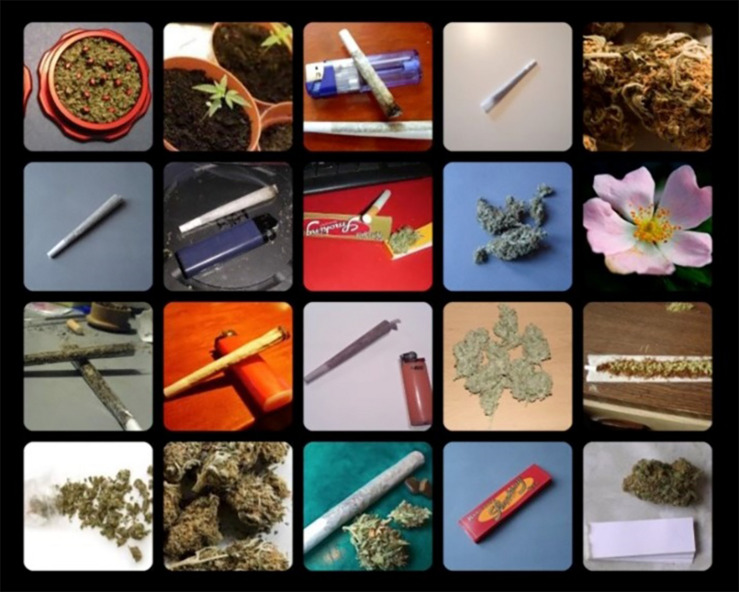
Example of a cannabis distractors trial of the OOOT.

Attentional bias toward alcohol- and cannabis-relevant cues was assessed using the OOOT (Rinck et al., 2005; Heitmann et al., 2017, 2020). In this task participants indicated whether there was a deviant stimulus (i.e., odd-one-out) among several distractors. Participants were first instructed to focus their attention on a fixation cross in the center of the screen (500 ms), after which a matrix of 4 × 5 images appeared. Responses about whether or not an odd-one-out was present were given by pressing the “0” (no odd-one-out present) or “1” (yes, odd-one-out present) button of the keyboard. There was a maximum of 10 s to respond, and participants were instructed to answer as quickly and accurately as possible. If present, the odd-one-out randomly appeared over the possible positions, but never directly above or below the fixation cross. The task was divided into three blocks of 24 trials each, which were randomly presented. The number of trials including an odd-one-out was based on the original task (Hansen and Hansen, 1988), whereas the number of trials without an odd-one-out (i.e., trials that are not critical for computing the bias indices) was reduced to minimize the burden for the participants, thereby also enhancing the feasibility of the clinical trial. In total, the task included three distinct categories of stimuli. That is, each image of the alcohol version of the OOOT belonged to one of the following three categories: alcoholic drinks, non-alcoholic drinks, or flowerpots. The cannabis version included images of cannabis use-relevant objects, neutral daily devices, and flowers. The contrast categories were chosen, because of their perceptually similar appearance with the substance-relevant stimulus categories. All images of the OOOT were used in previous studies with similar tasks (Pronk et al., 2015; van Hemel-Ruiter et al., 2016; Heitmann et al., 2017, 2020; see for example, Figures 1, 2). The task consisted of nine different trial types, three of which did not include an odd-one-out, whereas the other six trial types did so (see Table 1 for an overview of the trial types). In line with previous studies, correct responses on the six trial types including an odd-one-out were used to calculate the AB indices (e.g., Jonker et al., 2019b; Heitmann et al., 2020). That is, the detection index was calculated by subtracting the mean reaction time of the *alcohol/cannabis target trials* from the mean reaction time of the *neutral target in neutral distractors trials*. It is expected that if participants show speeded detection with substance-relevant cues, their attention would automatically be shifted toward a substance-relevant target presented among distractors resulting in a quicker response when compared to trials in which a neutral target is presented between neutral distractors. Thus, higher scores reflected more speeded detection of alcohol/cannabis cues. The distraction index was calculated by subtracting the mean reaction time of the *neutral target in neutral distractors trials* from the mean reaction time of the *alcohol/cannabis distractors trials*. Higher positive scores reflected more distraction by alcohol/cannabis cues.

**TABLE 1:** Type and number of trials in the Odd-One-Out task (OOOT).

Trial type			Trials per block
1.	Alcohol/cannabis-related objects (20)		2
2.	Non-alcoholic drinks/neutral daily devices (20)		2
3.	Flowerpots/flowers (20)		2

	**Target**	**Distractors**	

4.	Alcohol/cannabis-related object (1)	Non-alcoholic drinks/neutral daily devices (19)	3
5.	Alcohol/cannabis-related objects (1)	Flowerpots/flowers (19)	3
6.	Non-alcoholic drink/neutral daily device (1)	Alcohol/cannabis-related objects (19)	3
7.	Flowerpot/flower (1)	Alcohol/cannabis-related objects (19)	3
8.	Non-alcoholic drink/neutral daily device (1)	Flowerpots/flowers (19)	3
9.	Flowerpot/flower (1)	Non-alcoholic drinks/neutral daily devices (19)	3

*Number of presented images per trial is given in parentheses. Trial types 4 and 5 (i.e., alcohol target trials; cannabis target trials), trial types 6 and 7 (i.e., alcohol distractors trials; cannabis distractors trials), and trial types 8 and 9 (i.e., neutral target in neutral distractors trials) reflect the odd-one out trials and were included in the current analyses.*

### Procedure

#### Alcohol and Cannabis Group

Recruitment and data collection (April 2016 – February 2018) of outpatients of the *alcohol group* and *cannabis group* took place in the context of a multicenter randomized controlled trial (for more information see Heitmann et al., 2017), which was approved by the medical ethical committee of the University Medical Center of Groningen (METc 2016/026). The collection of the MATE-Q data took place during the intake procedure of regular addiction treatment. All other data (including the OOOT) were collected online during the baseline assessment of the trial using Qualtrics software. Participants first answered general questions and thereafter completed the OOOT. All participants provided written informed consent.

#### Comparison Groups

Data of the *alcohol comparison group* and the *cannabis comparison group* were collected between April 2017 and December 2018. Approval was given by the ethical committee of the psychology faculty of the University of Groningen (16265-O). Participants from the comparison groups were recruited via the network of the researchers, advertisement, and flyers. All data were collected online using Qualtrics software. Participants first gave their informed consent and then answered general questions (i.e., demographics). To be able to check for eligibility, all participants from the comparison groups were asked whether they ever received treatment for AUD/CUD, whether they were currently in treatment, or whether they think that they should search for help due to the amount of used alcohol/cannabis. Next, participants completed the OOOT and thereafter filled in the MATE-Q questions.

### Analyses

Group differences between the alcohol group and the alcohol comparison group on alcohol frequency, alcohol quantity, and age were assessed with independent samples *t*-tests. In line, group differences between the cannabis group and the cannabis comparison group on age and cannabis frequency were assessed with independent samples *t*-tests. Group differences between the alcohol group and the alcohol comparison group, and the cannabis group and the cannabis comparison group on gender were tested with a Chi-square independence test. To investigate differences between individuals diagnosed with AUD or CUD and adults without these diagnoses on AB indices, two between-groups multivariate analysis of variance (MANOVA) were performed with the two AB indices (detection and distraction) as dependent factor, and group (alcohol group and alcohol comparison group; cannabis group and cannabis comparison group) as fixed factor using IBM SPSS Statistics (IBM Corp, 2016, version 24.0). To increase confidence in our results delivered by the MANOVA following the frequentist approach, we also report results following the Bayesian approach. This part of the analysis was done in JASP (JASP Team, 2018, version 0.10.2.0). Given that to the best of our knowledge there is no option for a Bayesian MANOVA, we computed Bayesian independent samples *t*-tests using the default prior setting for effect size with a zero-cantered Cauchy distribution with a value of 0.707. For tests that delivered significant results following the frequentist approach BF10 was reported, quantifying the evidence for the alternative hypotheses over the null hypotheses (patients with AUD/CUD show speeded detection of substance-relevant cues, and increased distraction by these cues than individuals without these diagnoses). Tests that delivered non-significant results were reported by BF01, quantifying the evidence for the null hypotheses over the alternative hypotheses (patients with AUD/CUD do not differ with individuals without these diagnoses with regard to speeded detection of and increased distraction by substance-relevant cues). In case the results delivered either a BF10 or a BF01 below one, we also reported the mathematically equivalent statement, respectively, BF01 or BF10. The reported Bayes factors were considered “no evidence” when having a value of 1 or lower, “anecdotal” between 1 and 3, “moderate” between 3 and 10, “strong” between 10 and 30, “very strong” between 30 and 100, and “extreme evidence” when having a value above 100 (Wagenmakers et al., 2017).

### Data Preparation and Reduction

#### Odd-One-Out Task – Alcohol

##### Alcohol group

Participants of the *alcohol group* scoring three SD’s below the mean percentage correct answers (<65.7%) were removed (*n* = 4), because high numbers of incorrect responses might indicate unserious participation. In line with Hollitt et al. (2010), as a next step, incorrect responses of the relevant trials (i.e., participants indicated that there was no odd-one-out although an odd-one-out was present) were excluded from the analyses (34.7%). Table 2 shows the percentage of errors made on the OOOT per trial type for all groups. No reaction times below 200 ms, which were considered anticipation errors, were found. Finally, outliers were calculated based on participants’ average response time per type of trial. Based on this step, there were no further outliers based on trials scoring three SD’s below or above participants’ average response time. After data preparation, for five participants of the *alcohol group* we could not calculate the AB indices as there were no correct trials on one or more of the trial types. Further, we identified one extreme univariate outlier which was excluded from the analysis. Therefore, the final sample of this group that was included in the analyses consisted of 53 participants. Internal consistency was first assessed by calculating the AB indices for the first and the second half of the OOOT. This revealed a Spearman–Brown coefficient of 0.36 for the detection index, and 0.44 for the distraction index. Second, internal consistency was assessed by alternately assigning trials to two subsets, with the first trial being randomly assigned to one of the two sets. The relationship between the first set and the second set of trials revealed a Spearman–Brown coefficient of 0.38 for the detection index, and 0.31 for the distraction index.

**TABLE 2:** Percentage of incorrect responses on the OOOT per trial type for all four groups.

Trial type	Cue description	Alcohol group (*n* = 53)	Alcohol comparison group (*n* = 60)	Cannabis group (*n* = 17)	Cannabis comparison group (*n* = 26)
Trials without an odd-one-out	20 alcohol/cannabis images	27.1	17.8	28.0	17.3
	20 non-alcoholic drinks/neutral daily devices images	20.6	22.7	25.6	51.2
	20 flowerpots/flower images	16.4	11.7	13.1	13.7
Alcohol/cannabis target trials	1 alcohol/cannabis image with 19 non-alcoholic drinks/neutral daily devices images	46.3	39.3	30.6	32.9
	1 alcohol/cannabis image with 19 flowerpots/flower images	22.2	17.9	17.9	21.8
Alcohol/cannabis distractors trials	1 non-alcoholic drink/neutral daily device image with 19 alcohol/cannabis images	47.3	56.3	42.1	55.6
	1 flowerpot/flower image with 19 alcohol/cannabis images	23.2	16.9	29.4	32.9
Neutral target in neutral distractors trials	1 non-alcoholic drink/neutral daily device image with 19 flowerpots/flower images	26.9	19.1	54.0	29.4
	1 flowerpot/flower image with 19 non-alcoholic drinks/neutral daily devices images	30.5	20.0	70.6	36.9

##### Alcohol comparison group

Following the same steps, the data of two participants from the alcohol comparison group were removed as they were scoring three SD’s below the mean percentage correct answers (<72.8%). Next, incorrect responses of relevant trials were excluded from the analyses (32.6%; see Table 2). Reaction times below 200 ms were deleted (six trials). No further outliers, based on trials scoring three SD’s below or above participants’ average response time were found. Finally, the calculation of AB indices appeared to be impossible for one participant due to the lack of correct neutral target in neutral distractors trials. There was no participant who indicated to be in need for treatment with regard to his/her alcohol use. Therefore, the analyses included 60 participants of the alcohol comparison group. Following a similar manner of calculating the internal consistency of the OOOT, the relationship between the first half and the second half of trials resulted in a Spearman–Brown coefficient of −0.09 for the detection index, and 0.23 for the distraction index. In line, when alternately assigning trials to one of two subsets, internal consistency was 0.10 and 0.53 for detection and distraction, respectively.

#### Odd-One-Out Task – Cannabis

##### Cannabis group

In the cannabis group, there were no participants scoring three SD’s below the mean percentage correct answers (<63.9%). As a next step, incorrect responses of relevant trials were excluded from the analyses (30.0%; see Table 2). There were no reaction times below 200 ms. there were also no further outliers based on trials scoring three SD’s below or above participants’ average response time. After data preparation, for 11 participants of the cannabis group no AB indices could be calculated, because of too many incorrect responses, especially on neutral target in neutral distractors trials. Therefore, the final sample of this group consisted of 17 participants. Internal consistency following the split-half approach revealed a Spearman–Brown coefficient of 0.57 for the detection index, and 0.46 for the distraction index. Following the approach in which trials were alternately distributed to one of two subsets, internal consistency was 0.40 for the detection index, and 0.63 for the distraction index.

##### Cannabis comparison group

There were no participants in the cannabis comparison group who scored three SD’s below the mean percentage correct answers (<67.0%). Incorrect responses of relevant trials were removed (35.8%; see Table 2), and thereafter trials with reaction times below 200 ms were deleted (seven trials). No further outliers based on trials scoring three SD’s below or above participants’ average response time were found. Finally, there was one participant for which no AB indices could be calculated, because of too many incorrect responses on cannabis distractors trials. Further, one participant of this group was excluded as he/she indicated being currently in treatment for problems with regard to cannabis use. Therefore, the final sample of this group that was included in the analyses consisted of 26 participants. Internal consistency of the OOOT was −0.44 for the detection index, and 0.21 for the distraction index when following the split-half approach. When alternately distributing trials to one of two subsets Spearman–Brown coefficient was −0.10 for the detection index, and 0.57 for the distraction index.

#### Power Calculation

After the data preparation and reduction, based on power analysis for two-group independent sample *t*-tests, for the *alcohol group* (*n* = 53) and the *alcohol comparison group* (*n* = 60) the power to find a medium effect size of 0.5 at an alpha of 0.05 was 0.75, and a power to find a large effect size of 0.8 was 0.99; for the *cannabis group* (*n* = 17) and *cannabis comparison group* (*n* = 26) the power was 0.35 to find a between group difference of a medium effect size of 0.5 at an alpha of 0.05, and a power of 0.71 to find a between group difference of a large effect size of 0.8.

## Results

### Group Characteristics

The group characteristics of the four groups with regard to age, alcohol/cannabis frequency, and alcohol quantity are shown in Table 3. As can be seen, the *alcohol group* and the *cannabis group* did not differ with their comparison groups on age. Supporting the validity of the selection of participants, the *alcohol group* drank significantly more frequent, and higher amounts of alcohol than the *alcohol comparison group*. In line, the *cannabis group* used cannabis significantly more often throughout the past 30 days than the *cannabis comparison group*. From the *alcohol group* 58.5% of the participants were male, and 56.7% of the participants of the *alcohol comparison group* were male [χ(1) = 0.038, *p* = 0.845]. There were 76.5% male participants in the *cannabis group*, and 65.4% in the *cannabis comparison group* [χ(1) = 0.599, *p* = 0.439]. For further descriptives, such as marital status and level of education, see Table 4.

**TABLE 3:** Age, frequency, and quantity of substance use of all four groups.

	Alcohol group (*n* = 53)		Alcohol comparison group (*n* = 60)					Cannabis group (*n* = 17)		Cannabis comparison group (*n* = 26)				
	*M*	SD	*M*	SD	*t*	*p*	*d*	*M*	SD	*M*	SD	*t*	*p*	*d*
Age	49.55	11.91	48.70	13.36	0.35	0.724	0.07	30.53	5.14	33.50	8.64	−1.28	0.209	0.42
Frequency	16.91	11.91	9.27	9.23	3.84	<0.01	0.72	23.75	9.97	1.27	5.89	9.22	<0.01	2.75
Quantity	168.55	165.37	40.67	69.79	5.48	<0.01	1.01	–	–	–	–	–	–	–

*Frequency, number of days that participants have used alcohol/cannabis over the past 30 days; quantity, number of consumed standard units of alcohol during the past month.*

**TABLE 4:** Marital status and level of education of the alcohol group, the alcohol comparison group, the cannabis group, and the cannabis comparison group.

	Alcohol group (*n* = 53)	Alcohol comparison group (*n* = 60)	Cannabis group (*n* = 17)	Cannabis comparison group (*n* = 26)
	**%**	**%**	**%**	**%**
Marital status				
Unmarried	24.5	16.7	52.9	42.3
Married/living together	49.0	73.3	47.1	57.7
Divorced	24.5	8.3	–	–
Widowed	1.9	1.7	–	–
Level of education				
High school/university	24.5	30.0	17.6	65.4
Other secondary education	73.6	70.0	82.4	34.6
Other	1.9	–	–	–

### Descriptive Statistics

Per group, the mean reaction times for all three trial types (i.e., target trials, distractors trials, and neutral target in neutral distractors trials), as well as AB indices, which were calculated based on these types of trials, are presented in Table 5.

**TABLE 5:** Mean reaction times per trial type in ms and AB scores per group.

	Alcohol group (*n* = 53)		Alcohol comparison group (*n* = 60)		Cannabis group (*n* = 17)		Cannabis comparison group (*n* = 26)	
	*M*	SD	*M*	SD	*M*	SD	*M*	SD
Distractors trials	3977	1176	3744	1015	3890	763	3784	1117
Target trials	3339	903	3370	899	2871	401	3102	797
Neutral trials	3047	941	2847	851	3461	798	2980	824
Detection index	−292	620	−523	587	590	838	−122	542
Distraction index	930	789	896	828	429	1043	804	1040

*Neutral trials, neutral target in neutral distractors trials; detection index, neutral target in neutral distractors trials – alcohol/cannabis target trials; distraction index, alcohol/cannabis distractors trials – neutral target in neutral distractors trials.*

### Differences Between the Clinical and Comparison Groups on Attentional Bias Indices

#### Attentional Bias in Alcohol Use Disorder

Assumption testing for the MANOVA was performed to check for normality, linearity, univariate and multivariate outliers, homogeneity of variance-covariance matrices, and multicollinearity. No violations were found. The MANOVA showed a significant intercept [*F*(2,110) = 73.72, *p* < 0.001; Wilk’s Λ = 0.427, partial η2 = 0.57], indicating that overall AB indices differed from zero. Using a Bonferroni adjusted alpha level of 0.025, the detection index [*F*(1,111) = 50.48, *p* < 0.001, partial η2 = 0.31], as well as the distraction index [*F*(1,111) = 143.01, *p* < 0.001, partial η2 = 0.56] significantly differed from zero. Thus supporting its validity, the OOOT was sufficiently sensitive to detect differences in participants’ AB for neutral versus substance-relevant stimuli. Most important for the current context, the MANOVA revealed no significant differences with regard to the AB indices between the *alcohol group* and the *alcohol comparison group* [*F*(2,110) = 2.66, *p* = 0.074; Wilk’s Λ = 0.954, partial η2 = 0.05]. However, there appeared to be a trend in the expected direction, indicating that individuals with AUD tended to be faster in detecting the alcohol cues than individuals without this diagnosis. Following the Bayesian approach, we examined the BF_01_ to test the evidence in favor of the null hypothesis over the alternative hypothesis of no difference between the groups on detection and distraction. With regard to the detection index, the evidence for the null hypothesis appeared to be weak, with a BF_01_ of 0.43. The BF_10_, testing the evidence in favor of the alternative hypothesis over the null hypothesis (individuals with AUD show faster detection of alcohol cues than individuals without this diagnosis), revealed anecdotal evidence (2.35). Thus, the evidence with regard to speeded attentional detection of alcohol cues can be considered inconclusive. With regard to the distraction index, we found moderate evidence in favor of the null hypothesis with a BF_01_ of 4.18, suggesting that individuals with AUD did not show more distraction by the alcohol cues than individuals without this diagnosis.

#### Attentional Bias in Cannabis Use Disorder

For the second MANOVA, there were no violations of the assumptions. This MANOVA showed a significant intercept [*F*(2,40) = 26.25, *p* < 0.001; Wilk’s Λ = 0.432, partial η2 = 0.57], indicating that overall the AB indices differed from zero. Using a Bonferroni adjusted alpha level of 0.025, the distraction index [*F*(1,41) = 14.41, *p* < 0.001, partial η2 = 0.26] significantly differed from zero, whereas the detection index [*F*(1,41) = 4.96, *p* = 0.031, partial η2 = 0.11] did not. Further and more importantly in the context of the current study, there was a statistical significant difference between the cannabis group and the cannabis comparison group on the combined dependent variables [*F*(2,40) = 6.62, *p* = 0.003; Wilk’s Λ = 0.751, partial η2 = 0.25]. Using a Bonferroni adjusted alpha level of 0.025, subsequent univariate tests indicated that only attentional detection of cannabis cues was found to be significantly different between groups [*F*(1,41) = 11.52, *p* = 0.002, partial η2 = 0.22]. As expected, an inspection of the mean scores indicated that individuals with CUD faster detected cannabis-relevant cues (*M* = 590.13, SD = 838.22) than individuals without this diagnosis (*M* = −122.38, SD = 541.65). Following the Bayesian approach, the evidence for a difference between the two groups on facilitated detection was very strong, with a BF_10_ of 43.48, suggesting that individuals with CUD indeed showed speeded detection of cannabis cues when compared to individuals without this diagnosis. With regard to distraction, there was moderate evidence that individuals with CUD did not differ from individuals without this diagnosis with regard to distraction by cannabis cues, as indicated by a BF_01_ of 6.24.

## Discussion

The current study used an Odd-One-Out visual search task (OOOT), to examine if individuals diagnosed with AUD or CUD are characterized by speeded detection of substance-relevant cues and/or increased attentional distraction by these cues. Therefore, scores on both AB indices were compared with scores of two age and gender matched community samples that had no treatment history of substance use disorder and were therefore considered non-addicted substance users. The study findings indicated no evidence for increased distraction from alcohol/cannabis cues in individuals with AUD and CUD when compared to individuals without this diagnosis. However, individuals with CUD showed faster detection of cannabis cues than individuals without this diagnosis. It remained inconclusive whether individuals with AUD are characterized by speeded detection of alcohol cues when compared to individuals without this diagnosis.

Attentional bias is suggested to play a critical role in the persistence of substance use disorders (Wiers et al., 2007; Gladwin and Figner, 2015), especially by the means of its proposed reciprocal relationship with craving (Franken, 2003; Field et al., 2009). However, the empirical evidence appears to be less straightforward (e.g., Field et al., 2016), which might be related to the configuration of previously used assessment tasks (i.e., inability to differentiate between detection and distraction components of attention, and a confined stimulus representation). Further, most studies that investigated the role of AB relied on non-clinical participants or clinical samples of very modest sample size (e.g., Field et al., 2009; Christiansen et al., 2015). To follow-up on previous studies, the current study examined the role of AB in two samples of clinically diagnosed treatment seeking individuals, of which the AUD sample was relatively large (sufficient to reliably detect also differences of medium effect size), using a challenging multi-stimulus assessment task delivering two separate indices of AB for detection and distraction bias.

With regard to AUD, we found a trend for the speeded detection component of AB showing that individuals with AUD might attend more quickly to alcohol-relevant cues than individuals without this diagnosis. However, the evidence was weak, and it should therefore be considered inconclusive whether or not individuals with AUD are characterized by speeded detection of alcohol-relevant cues. Of course, the inconclusive results might indicate that detection bias is not relevant in AUD. Another reason for the inconclusive results might be that the stimuli of one contrast category that has typically also been used in previous research (i.e., non-alcoholic drinks) were closely related to the category of interest (i.e., alcoholic drinks). As suggested by previous findings, individuals with AUD might show a tendency to faster attend to cues that are related to appetitive stimuli in general, and not to alcohol-relevant stimuli only (Wiers et al., 2009; Pennington et al., 2019; Qureshi et al., 2019). The visual similarity of the images of the alcoholic and non-alcoholic stimuli might have further lowered the sensitivity to find a convincing difference in AB between the clinical and the comparison group. In line, the visual similarity might also help explain the high number of incorrect responses that have been made throughout the task, which in turn might have reduced the sensitivity of the current task as a measure of AB (Ataya et al., 2012). One way to further investigate the role of speeded detection of alcohol-relevant cues (i.e., initial orientation) in AUD would be to test an adapted version of the OOOT, including two contrast categories that can be considered more neutral/unrelated to critical features of the alcohol stimuli and therefore more distinct from the alcohol stimuli.

By using the OOOT, we further found that distraction by alcohol-relevant cues does not seem to characterize individuals diagnosed with AUD. That is, no difference was found between individuals with AUD and individuals without this diagnosis in their extent of being distracted by alcohol-relevant cues. In particular, as indicated by positive means, which significantly deviated from, zero, both groups seemed to have a tendency of being more distracted by alcohol cues than by neutral cues. More distraction by alcohol cues might therefore be a general feature of individuals drinking alcohol on a regular basis, and not a specific characteristic of individuals with AUD. This finding is in line with results of a previous study showing that increased distraction was related with alcohol consumption in a non-clinical student sample (Heitmann et al., 2020). Together these findings might suggest that increased distraction plays no relevant role in the persistence of addiction. However, when trying to quit alcohol, having a tendency of being more distracted by alcohol cues than by neutral cues might nevertheless be problematic. Future studies might therefore further investigate whether being relatively strongly distracted by alcohol cues has negative influences on treatment outcome in AUD.

With regard to CUD, the results showed that individuals diagnosed with CUD were faster in detecting cannabis cues than individuals without this diagnosis. This speeded detection might contribute to the persistence of addictive behavior in CUD. Having a heightened tendency to detect cues that are related to cannabis use might heighten the probability of cue-triggered urge to use (Franken, 2003; Field et al., 2009), and thereby maintaining a bias-craving-bias cycle. In order to further investigate to what extent speeded detection of cannabis-relevant cues contributes to the persistence of CUD, and thereby may complicate the attempt to quit, it seems relevant to examine whether modifying this attentional tendency can positively contribute to treatment outcome (see for example, Heitmann et al., 2017).

Further, the current findings suggested that attentional distraction by cannabis-relevant cues does not specifically characterize individuals with CUD, as no difference was found when compared with individuals without this diagnosis. However, in line with the results in AUD, both groups showed a tendency for increased distraction by cannabis-relevant cues when compared with neutral cues. Although this tendency might not be specific for individuals with CUD, it might nevertheless be problematic once individuals with CUD decide to stop the use of cannabis. That is, maintaining attention on cannabis-relevant cues might contribute to the reciprocal relationship with craving (Franken, 2003; Field et al., 2009). Training individuals with CUD to strengthen their ability to quickly disengage attention from cannabis-relevant cues might therefore positively contribute to treatment outcome. However, it might also be that these findings suggest that distraction bias does not contribute to the persistence of addiction, as it is less relevant in problematic and clinical substance use. As a next step, it seems relevant to further investigate the causal influence of attentional distraction by cannabis-relevant cues on the persistence of CUD, for example by means of training to resist distractors (e.g., Cox et al., 2015), and investigating the effects on treatment outcome.

Overall, the current findings suggest that differentiating between detection and distraction bias might be relevant in the context of addiction as both biases might be independently (and differentially) involved in (non-)addictive behavior. In particular, the current results suggest that speeded detection is involved in addictive behavior and might maintain the proposed bias-craving-bias cycle, whereas increased distraction by substance-relevant cues seems less relevant. However, it is important to consider that a heightened tendency of becoming distracted by substance-relevant cues might perhaps be only expressed under certain circumstances which may not have been captured in the current study. Within the context of eating disorder problems it has been speculated that distraction bias may only arise when people are high in craving for food (Jonker et al., 2019b). In line, one could argue that also within the context of AUD or CUD, heightened distraction may only be evident when people experience strong craving for alcohol or cannabis. It might therefore be interesting for future research to assess AB following a craving induction procedure. Generally, it seems important that future research takes motivational and environmental factors into account when assessing AB (e.g., Heitmann et al., 2021), as AB has been suggested to have state like properties that can be influenced by several factors, such as the perceived availability of the substance or the time of the day (e.g., Field et al., 2013; Christiansen et al., 2015).

This study has several strengths, such as the inclusion of clinical samples of both individuals diagnosed with AUD and CUD, the usage of a task with a complex task configuration that is at the same time able to differentiate between two components of AB, and the assessment of AB in a substance use-relevant context (i.e., the home environment) rather than in a lab-context which might be associated with limited availability of the substance possibly influencing the ecological validity of the AB assessment (e.g., Droungas et al., 1995; Dols et al., 2000, 2002; Carter and Tiffany, 2001). The current study has also some limitations. First, in all four groups we found a high number of incorrect responses in the OOOT. Because the AB measures rely on accurate trials this reduced the available data points for extracting the AB indices, which in turn might reduce the sensitivity of the indices as measures of AB. One explanation for the relatively many errors compared to previous research using the OOOT (cf. Jonker et al., 2019b) was the use of a contrast category (i.e., non-alcoholic drinks) which was visually and content-wise relatively similar to the target category (i.e., alcoholic drinks). It could therefore be relevant for future research to use contrast categories that are more distinct from the alcohol stimuli. Second, although the online assessment of AB in the home environment can be considered a strength because AB was measured in a relevant context, it may also have contributed to the high error rate as in the home environment there is less control and possibly more distraction. This would also help explain why in the current study more errors were made than in a previous study using the same task but assessing AB in a laboratory setting (Heitmann et al., 2020). In line, assessing AB in the home environment does not allow to standardize the materials used by the participants (e.g., different computer screens/laptops). Third, individuals diagnosed with AUD and CUD were matched with the two comparison groups based on age and gender. There was no control on possible other differences between the groups. As derived from the descriptives, individuals with CUD and individuals from the comparison group might differ in their level of education. Although there are no obvious other differences between these two groups, for example in their performance on the OOOT (number of incorrect responses), level of education might nevertheless have affected the results and should therefore be kept in mind when interpreting the results. Fourth, the study did not include other measurements of substances, for example tobacco, which might have influenced especially the measures of the cannabis and the cannabis comparison group due to its visual similarities with the cannabis-relevant cues. Future studies might want to control for possible interfering effects. Fifth, the sample of individuals diagnosed with CUD and its comparison group were rather small. It would therefore be important to test in future research whether the present findings are replicable and robust. Sixth, for all four groups the internal consistency of the OOOT was found to be lower than the conventional threshold of 0.7. Although low reliability of the AB measures is less critical within the current between group design, it does question the relevance of the OOOT as a measure of individual differences, for example in prospective designs.

## Conclusion

To conclude, the findings of the current study give a first indication that distinguishing between speeded detection and increased distraction might be relevant when assessing AB in substance use disorders. The current study found no support for the view that individuals diagnosed with AUD or CUD are characterized by an increased distraction by substance-relevant cues when compared with individuals without these diagnoses. The findings did, however, support the view that CUD is associated with speeded detection of cannabis cues which might help explain the persistence of CUD; although a similar trend was evident for AUD, the evidence was weak and remained therefore inconclusive. Given the high error rates of the current task and related low reliability of the current AB measures, future research should further investigate the differential role of both attentional processes.

## Data Availability Statement

The raw data supporting the conclusions of this article will be made available by the authors, without undue reservation.

## Ethics Statement

The studies involving human participants were reviewed and approved by the Medical Ethical Committee of the University Medical Center of Groningen and by the Ethical Committee of Psychology (ECP), University of Groningen. The patients/participants provided their written informed consent to participate in this study.

## Author Contributions

JH and PJ designed the study. JH was responsible for the data collection and analysis, and drafted this manuscript. Both authors further contributed to the writing process and approved this final manuscript.

## Conflict of Interest

The authors declare that the research was conducted in the absence of any commercial or financial relationships that could be construed as a potential conflict of interest.
